# Multi-omics analyses reveal aberrant differentiation trajectory with WNT1 loss-of-function in type XV osteogenesis imperfecta

**DOI:** 10.1093/jbmr/zjae123

**Published:** 2024-08-10

**Authors:** Zhijia Tan, Peikai Chen, Jianan Zhang, Hiu Tung Shek, Zeluan Li, Xinlin Zhou, Yapeng Zhou, Shijie Yin, Lina Dong, Lin Feng, Janus Siu Him Wong, Bo Gao, Michael Kai Tsun To

**Affiliations:** Department of Orthopaedics and Traumatology, The University of Hong Kong-Shenzhen Hospital, Shenzhen, 518053, China; Clinical Research Center for Rare Diseases, The University of Hong Kong-Shenzhen Hospital, Shenzhen, 518083, China; Department of Orthopaedics and Traumatology, Li Ka Shing Faculty of Medicine, The University of Hong Kong, Hong Kong, China; Department of Orthopaedics and Traumatology, The University of Hong Kong-Shenzhen Hospital, Shenzhen, 518053, China; Clinical Research Center for Rare Diseases, The University of Hong Kong-Shenzhen Hospital, Shenzhen, 518083, China; School of Biomedical Sciences, Li Ka Shing Faculty of Medicine, The University of Hong Kong, Hong Kong, China; The AI and Big Data Lab, The University of Hong Kong-Shenzhen Hospital, Shenzhen, 518053, China; Department of Orthopaedics and Traumatology, Li Ka Shing Faculty of Medicine, The University of Hong Kong, Hong Kong, China; School of Biomedical Sciences, Faculty of Medicine, The Chinese University of Hong Kong, Hong Kong, China; Department of Orthopaedics and Traumatology, The University of Hong Kong-Shenzhen Hospital, Shenzhen, 518053, China; Clinical Research Center for Rare Diseases, The University of Hong Kong-Shenzhen Hospital, Shenzhen, 518083, China; Department of Orthopaedics and Traumatology, The University of Hong Kong-Shenzhen Hospital, Shenzhen, 518053, China; Clinical Research Center for Rare Diseases, The University of Hong Kong-Shenzhen Hospital, Shenzhen, 518083, China; Department of Orthopaedics and Traumatology, The University of Hong Kong-Shenzhen Hospital, Shenzhen, 518053, China; Department of Orthopaedics and Traumatology, The University of Hong Kong-Shenzhen Hospital, Shenzhen, 518053, China; Clinical Research Center for Rare Diseases, The University of Hong Kong-Shenzhen Hospital, Shenzhen, 518083, China; Department of Orthopaedics and Traumatology, The University of Hong Kong-Shenzhen Hospital, Shenzhen, 518053, China; Clinical Research Center for Rare Diseases, The University of Hong Kong-Shenzhen Hospital, Shenzhen, 518083, China; Department of Orthopaedics and Traumatology, The University of Hong Kong-Shenzhen Hospital, Shenzhen, 518053, China; Clinical Research Center for Rare Diseases, The University of Hong Kong-Shenzhen Hospital, Shenzhen, 518083, China; Department of Orthopaedics and Traumatology, The University of Hong Kong-Shenzhen Hospital, Shenzhen, 518053, China; Clinical Research Center for Rare Diseases, The University of Hong Kong-Shenzhen Hospital, Shenzhen, 518083, China; Department of Orthopaedics and Traumatology, The University of Hong Kong-Shenzhen Hospital, Shenzhen, 518053, China; Clinical Research Center for Rare Diseases, The University of Hong Kong-Shenzhen Hospital, Shenzhen, 518083, China; Department of Orthopaedics and Traumatology, Li Ka Shing Faculty of Medicine, The University of Hong Kong, Hong Kong, China; Department of Orthopaedics and Traumatology, The University of Hong Kong-Shenzhen Hospital, Shenzhen, 518053, China; Clinical Research Center for Rare Diseases, The University of Hong Kong-Shenzhen Hospital, Shenzhen, 518083, China; School of Biomedical Sciences, Faculty of Medicine, The Chinese University of Hong Kong, Hong Kong, China; Department of Orthopaedics and Traumatology, The University of Hong Kong-Shenzhen Hospital, Shenzhen, 518053, China; Clinical Research Center for Rare Diseases, The University of Hong Kong-Shenzhen Hospital, Shenzhen, 518083, China; Department of Orthopaedics and Traumatology, Li Ka Shing Faculty of Medicine, The University of Hong Kong, Hong Kong, China

**Keywords:** Type XV Osteogenesis imperfecta, WNT1, Proteomics, Single-cell transcriptomics

## Abstract

Osteogenesis imperfecta (OI) is a group of severe genetic bone disorders characterized by congenital low bone mass, deformity, and frequent fractures. Type XV OI is a moderate to severe form of skeletal dysplasia caused by *WNT1* variants. In this cohort study from southern China, we summarized the clinical phenotypes of patients with *WNT1* variants and found that the proportion of type XV patients was around 10.3% (25 out of 243) with a diverse spectrum of phenotypes. Functional assays indicated that variants of *WNT1* significantly impaired its secretion and effective activity, leading to moderate to severe clinical manifestations, porous bone structure, and enhanced osteoclastic activities. Analysis of proteomic data from human skeleton indicated that the expression of SOST (sclerostin) was dramatically reduced in type XV patients compared to patients with *COL1A1* quantitative variants. Single-cell transcriptome data generated from human tibia samples of patients diagnosed with type XV OI and leg-length discrepancy, respectively, revealed aberrant differentiation trajectories of skeletal progenitors and impaired maturation of osteocytes with loss of WNT1, resulting in excessive *CXCL12^+^* progenitors, fewer mature osteocytes, and the existence of abnormal cell populations with adipogenic characteristics. The integration of multi-omics data from human skeleton delineates how WNT1 regulates the differentiation and maturation of skeletal progenitors, which will provide a new direction for the treatment strategy of type XV OI and relative low bone mass diseases such as early onset osteoporosis.

## Introduction

Osteogenesis imperfecta (OI) is a group of inherited skeletal dysplasia, with the prevalence of 0.3–0.7 per 10 000 births.^1^ Individuals with OI are characterized with low bone mass and high bone fragility, resulting in susceptibility to long bone deformity, fracture, and vertebral compression.^2^ A wide spectrum of secondary features may be present, including blue sclerae, dentinogenesis imperfecta, scoliosis, hearing loss, muscle weakness, ligamentous laxity, and basilar invagination.^1^^,^^3^ The phenotypes and severities of OI are diverse, and clinically classified into 4 major types, including type I (mild with bone fragility and blue sclerae), type II (perinatal lethality), type III (progressive deformity), and type IV (between type I and type III with normal sclerae, short stature, bone deformity, and dentinogenesis imperfecta).^4^^,^^5^ Most patients are heterozygous for dominant variants in *COL1A1* or *COL1A2*, encoding the main components of extracellular matrix in bone and skin. Recent genetic analyses have identified numerous variants in genes involved in post-translational modification and processing of type I collagen, bone mineralization, and osteoblast differentiation. Of note, type XV OI, first reported in 2013 to be affected by recessive variants on *WNT1,*^6^ represents 5%–10% in many Asian cohorts.^7^^,^^8^

Although WNT1 was originally identified as an essential regulator for midbrain and cerebellum development,^9^ several research groups reported recently that homozygous variants in *WNT1* caused moderate-to-severe OI syndromes in patients, and heterozygous *WNT1* variants resulted in early onset of osteoporosis,^6^^,^^10^^-^^12^ suggesting WNT1 as a key regulator for bone homeostasis. In mice, WNT1 interacts with Frizzled receptor and co-receptor LRP5/6 to modulate bone formation. Deletion of *Wnt1* by Dmp1-Cre in osteocytes resulted in low bone mass with spontaneous fractures. Conversely, Wnt1 overexpression in osteocytes stimulated bone formation via increasing osteogenic activity.^13^ Despite these studies using different *Wnt1* mutant models,^13^^-^^15^ the exact molecular mechanism and cellular changes underlying type XV OI remain unclear.

In this article, we summarized the mutational spectrums of *WNT1* and clinical phenotypes in a Chinese cohort of type XV OI patients. We investigated the impacts of the variants on WNT1 activities and applied human proteomic and single-cell transcriptome to further explore the differentiation trajectory of osteogenic progenitors and the downstream pathways regulated by WNT1, aiming to uncover the pathogenic mechanisms of type XV OI and provide a new direction for the treatment strategy.

## Materials and methods

### Ethical compliance

This study was approved by the Institutional Review Broad of HKU-Shenzhen Hospital ([2016]08 and [2020]190). Detailed medical features were collected by clinicians. Peripheral blood was obtained for genetic test. Available skeletal tissues discarded after operations were collected for histological analyses, proteomics, and single-cell RNA sequencing. Informed consent was obtained for all patients and their parent(s)/ guardian(s) if the patients were younger than 16 yr old.

### Targeted amplicon sequencing

DNA from peripheral blood was subjected to targeted amplicon sequencing as described previously.^16^^,^^17^ Libraries were amplified and incorporated with a unique 8-bp index before sequencing on the NovaSeq 6000 system (Illumina) with a 150 bp paired-end protocol in Dynasty Gene Company and aligned to GRCh37/hg19 human genome. The GATK toolkit^18^ was applied to call the variants and annotated by SNPeff^19^ and ANNOVAR.^20^ Twenty OI causative genes and 5 OI-related genes were included in the sequencing panel.^16^^,^^17^

### Mutagenesis and minigene splicing assay

The full-length coding sequence of human *WNT1* was cut from pcDNA-Wnt1-V5 plasmid (Addgene, #35924) and inserted into pcDNA3.1 plasmid. For minigene splicing assay, WT *WNT1* genomic DNA fragment containing exon1, intron 1, and exon2 (1817 bp) was also cloned into the pcDNA3.1 plasmid. The 12 exonic variants and 1 intronic variant (c.104+1G>A) were introduced by PCR-based site-directed mutagenesis and verified by Sanger sequencing. Purified constructs were transfected into HEK293T cells using Polyethylenimine. RNA was extracted 24 h after transfection. Splicing patterns were tested by specific primers (primer F: 5′-GGCAACAACCAAAGTCGCC-3′, primer R: 5′-CCCCGGATTTTGGCGTATCA-3′) and verified by Sanger sequencing.

### Western blot

MC3T3-E1 cells cultured with DMEM were transfected with *WNT1* overexpression vectors (WT or mutants, 2 μg/well) using Lipofectamine 3000 (Thermo Fisher). Cells were lysed with RIPA buffer 24 h after transfection, and the corresponding medium was collected for protein enrichment using StrataClean Resin (Agilent). In brief, 20 μL aliquots of beads were primed by incubation with 12M HCl at 100°C for 5 h before applying for protein binding. After incubation with 2 mL complete cultured medium overnight at 4°C, the resin beads were washed 5 times with TE buffer and sedimented by centrifugation at 8000*g*. Enriched secreted proteins were mixed with protein loading buffer and boiled at 100°C for elution. Western blots were conducted using rabbit anti-WNT1 (A2475, ABclonal), mouse anti-GAPDH (AC002, ABclonal), and rabbit anti-fibronectin (1:1000, A12932, ABclonal) antibodies.

### Dual luciferase reporter assay

HEK293T cells were seeded in a 24-well plate (5 × 10^4^ cells/well) and transfected with WT or mutant *WNT1* plasmids (500 ng/well). Conditional medium was collected 24 h after transfection. Another batch of HEK293T cells was prepared for transfection with M50 Super 8x TOPFlash plasmid (20 ng/well, Addgene, #12456) and renilla (10 ng/well, Addgene, #118059). After 6 h, 300 μL conditional medium was refilled into each well for treatment. Cells were harvested 24 h after treatment and detected with Dual-Glo® luciferase assay system (Promega).

### Quantitative RT-PCR

MC3T3-E1 cells were transfected with WT or mutant *WNT1* plasmids and cultured with osteogenic medium (αMEM, 50 μg/mL ascorbic acid, 10 mM β-glycerophosphate) for 4 d. Then RNA was extracted and TB Green Master Mix (TaKaRa) was used for qRT-PCR. Specific primers were designed as following: mouse *Axin2* (forward 5′-CCAGGCTGGAGAAACTGAAA-3′; reverse 5′-AGAGGTGGTCGTCCAAAATG-3′) and *Gapdh* (forward 5′-GTGTTTCCTCGTCCCGTAGA-3′; reverse 5′-GAATTTGCCGTGAGTGGAGT-3′). Transcription level of *Axin2* was normalized to *Gapdh* level (*n* = 5).

### Statistics

Data were presented as averages with SD. Statistical significance level was evaluated by Student’s *t*-test (2-tailed, unpaired) between 2 groups. The difference with *p*<.05 was considered to be significant. The statistical methods used in the analyses of proteomic and scRNA-seq data are described in a later section.

### Histological analysis

The specimens were fixed in 4% paraformaldehyde and decalcified with 0.5M EDTA before embedding in paraffin. About 6 μm sections were cut and mounted on glass slides (Thermo Fisher). The rehydrated sections were stained with Goldner’s trichrome (Beyotime Biotechnology) as previous description.^21^

### Tartrate-resistant acid phosphatase staining

Tartrate-resistant acid phosphatase (TRAP) staining was performed as previously described.^22^ Basic incubation medium (pH 4.7–5.0) was freshly prepared (100 mM sodium acetate, Sigma; 50 mM sodium tartrate, Sigma; and 0.28% vol/vol acetic acid, Aladdin). Rehydrated sections were incubated in 50 mL basic medium with 0.5 mL Naphthol AS-BI phosphate (Sigma) solution (2% wt/vol in dimethyl formamide, Aladdin) at 37°C for 1 h. Nitrite-pararosaniline solution was freshly prepared by mixing equal volume of sodium nitrite solution (4% sodium nitrite, Sigma) and pararosaniline dye (5% pararosaniline dye in 2N HCL, Sigma). Slides were transferred into development solution (4 mL nitrite-pararosaniline solution into 100 mL basic medium) for 5 min at 37°C before counterstaining with Mayer’s hematoxylin.

### Immunofluorescent staining

Immunofluorescent staining was performed as previously described.^23^ Slides were dewaxed and rehydrated before blocking with blocking buffer (5% donkey serum (Jackson ImmunoResearch), 0.1% Triton X-100 (Sigma) in PBS) for 1 h. Samples were incubated with primary antibodies (rabbit anti-PERILIPIN, Cell Signaling, 9349S; goat anti-SOST, R&D, AF1589) overnight at 4°C and then detected with Alexa Fluor 488 secondary antibody (donkey anti-rabbit, donkey anti-goat, Thermo Fisher) and mounting medium with DAPI (Vector Laboratories). Images were taken under the AXIO Imager M2 microscope using ZEN 3.2 platform (Zeiss).

### Proteomic analysis of human bone tissues

The bone tissues in the tibia/femur diaphysis regions were collected after osteotomies from 3 individuals in the control group (OI patients with variants in *COL1A1*: c.858+1G>A, male, 14 yr old; c.4014T>A, p.Tyr1338^*^, biological duplicate, male, 12 yr old) and 3 patients diagnosed with type XV OI (*WNT1*: c. 619C>T, homozygous, female, 23 yr old; c.677C>T, c.877G>A, female, 9 yr old; c.121_122del, c. 385G>A, male, 2 yr old). The patients with *COL1A1* (c.858+1G>A) and *WNT1* (c.677C>T, c.877G>A) variants received bisphosphonate treatments, which were ceased at least 3 mo before and after the osteotomies. Soft tissues were totally removed and skeletal samples were pulverized into powder in liquid nitrogen for protein extraction before sending for nanoLC-MS/MS (Q Exactive HF-X and EASY-nLC 1200) mass spectrometry at Sangon Biotech. The data were acquired in data-independent acquisition (DIA) mode, whereby each scan cycle consisted of 1 MS1 scan (scan range 350–1250 m/z, resolution 60 K, AGC 3e6, max. IT = 30 ms) and 40 variable window MS2 scans (resolution 15 K, AGC 1e6, max. IT = 50 ms). The DIA raw data files were processed with the DIA-NN software (v 1.8),^24^ using the human proteome reference database on UniProt (dated March 12, 2021, total 20 381 protein sequences). Final results were screened for precursor and protein levels at 1% FDR. Protein-level case–control comparisons were performed using *t*-testing with a significance threshold of *p*<.05.

### Isolation of cells for single-cell transcriptomics

The skeletal tissues in the mid-shaft of the tibia diaphysis regions (1–2 cm) involving periosteum and endosteum were collected after tibia osteotomies from a patient (male, 8 yr old) carrying compound heterozygous variants on *WNT1* (c. 371C>T and c. 620G>A) and a patient with leg-length discrepancy (LLD) (male, 7 yr old) (Figure S1). The type XV OI patient received biannual bisphosphonate treatments since 2018. The BMD *Z*-scores were recorded in the FN regions (July 2018: −5.5; August 2019: −4.7; July 2020: −4.6). No bisphosphonate was delivered at least 3 mo before and after osteotomies. The control patient was diagnosed with Proteus syndrome, with genetic testing confirming a mosaic variant (c.49G>A, p.E17K, sample frequency 13.3%) in the *AKT1* gene. Single-cell RNA-seq reads happened to cover this region, where it showed a frequency of 6.3% (5 out of 79).

Single-cell RNA sequencing was conducted as previously described.^25^ Soft tissues and blood cells were fully removed with brief TrypLE Express digestion (Gibco). Then bone tissues were cut into fragments and digested with 0.25% Dispase, 0.25% type II collagenase in HBSS (Gibco) at 37°C on a shaker for 1.5 h. During the digestion, cells were collected every 30 min interval and filtered with a 40 μm cell strainer. Red blood cells were removed using Red Blood Cell Lysis Solution (Miltenyi Biotec) and cleaned up with Dead Cell Removal Kit (Miltenyi Biotec). Single cell suspension was washed twice with DPBS (Gibco). Viabilities of 85% and 82% were recorded for the LLD and type XV OI samples, respectively. A raw total input of 10 000 cells was estimated for each 10× sequencing. Single-cell encapsulation and library preparation were prepared by Chromium single-cell platform (10x Genomics Inc.) in Berry Genomics Co. Single cells were then encapsulated into GEMs by 10× Chromium Controller. Single-Cell 3′ Reagent Kits v3 was used for library construction according to the manufacturer’s protocol. The double-stranded cDNA went through enzymatic fragmentation, adapter ligation, index PCR, and SPRIselect size selection. Library size and concentration were determined by Qubit and Bioanalyzer assays.

### Single-cell RNA sequencing and bioinformatics analyses

Single-cell sequencing using 10× was performed on the Illumina NovaSeq 6000 platform at Berry Genomics Co., with 150 GBp throughput per sample. The raw data were aligned to human genome (GRCh38/hg38). Cells with fewer than 1600 genes or clusters expressing *PTPRC* (*CD45*) were excluded. Data were further processed with Seurat (v3.9.9). Population signatures were identified by comparing every population against all other cells, using Seurat’s FindAllMarkers function, with an FDR cutoff of <0.05, log2(fold-change)>1, and expressed percentage point difference >25%. The osteogenic cells were integrated using the canonical correlation analysis approach. Gene ontology analyses were performed using GSEA (gsea-msigdb.org).

### Bulk RNA sequencing

Human bone marrow MSCs were purchased from Cyagen Biosciences (HUXMF-01001). The cells were transfected with pcDNA3.1 and pcDNA3.1-*WNT1* and selected using G418 for 7 d*.* After expansion, the transfected MSCs (pcDNA3.1 and pcDNA3.1-*WNT1*) were lysed with Trizol (Thermo Fisher) for RNA extraction. RNA concentration was measured by Qubit, and RNA integrity was assessed using the Agilent 2100. A total amount of 2 μg RNA per sample was used for sequencing. Libraries were generated using VAHTS mRNA-seq Library Prep Kit and sequenced on an Illumina NovaSeq platform to generate 150 bp paired-end reads in Berry Genomics Co. (Beijing).

### Osteoblastic and adipogenic differentiation

Bone marrow MSCs transfected with pcDNA3.1-*WNT1* or empty vectors were treated with osteoblastic (aMEM with 10% FBS, 0.1 μM dexamethasone, 50 μg/mL ascorbic acid, and 10 mM β-glycerol phosphate) and adipogenic (DMEM with 10% FBS, 500 μM IBMX, 1 μM dexamethasone, 10 μg/mL insulin, and 1 μM Rosiglitazone) differentiation media for 14–28 d. The differentiated cells were fixed with 4% PFA and stained with Alizarin red (Sigma) or Oil red (Beyotime Biotechnology) kits according to the manufacturers’ protocols. The precipitated Alizarin red was dissolved using 10% acetic acid for 30 min. Then 10% ammonium hydroxide was added to neutralize the acid (acetic acid/ammonium hydroxide = 4:1). The solution was transferred to 96-well plates and the absorbance was read at the wavelength of 405 nm.

### Data availability

The transcriptomic sequencing data were deposited in the NCBI GEO website (Bulk RNA-seq: GSE262092; Single-cell RNA-seq: GSE262091). The proteomic data were deposited in the PRIDE database (www.ebi.ac.uk/pride) with accession number PXD050841. Scripts were deposited at https://github.com/HKUSZH/typeXVOI.

## Results

### Pathogenic variants detected in *WNT1* locus in our cohort

**Figure 1:**
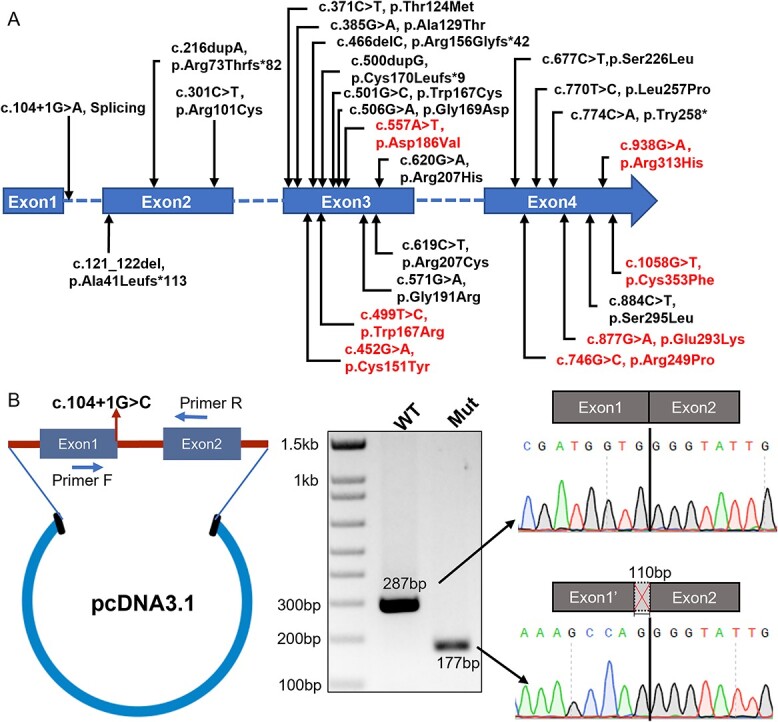
The mutational spectrum of *WNT1* identified in our OI cohort and minigene splicing assay. (A) Diagram showing the distribution of *WNT1* variants identified in our OI cohort. The *WNT1* gene consists of 4 exons with total of 24 variants indicated, including 19 reported variants (black) and 5 novel variants (red). (B) Construct of pcDNA3.1 vector used in minigene splicing assay (left panel) and validation of aberrant splicing caused by the variant c.104+1G>C using PCR and Sanger sequencing (right panel).

In the past 5 yr (2018–2023), 243 patients diagnosed with OI were recruited for genetic tests in our hospital.^21^ The panel sequencing involving 20 OI causative genes and 5 OI-related genes revealed that 25 patients (10.3%) harbored pathogenic variants in the *WNT1* locus, ranking as the most common recessive gene affecting OI in our cohort. Among them, 19 patients were compound heterozygous and 6 were homozygous (Table 1). Based on the databases of OI variants, dbSNP, ClinVar, and the 1000 Genomes project, all *WNT1* variants were classified as reported variants (black) and non-reported variants (red). The pathogenicity (pathogenic, likely pathogenic and variants of uncertain significance) of each variant was assessed according to the 2015 ACMG Standards and Guidelines.^26^ The locations of the variants were displayed in the *WNT1* locus (Figure 1A). Several recurrent variants were identified, including c.371C>T (5/25), c.501G>C (3/25), and c.677C>T (5/25), among which c.677C>T has been described as a hot-spot variant previously in a Chinese cohort study involving 20 type XV OI patients.^12^

**Table 1:** Identification of *WNT1* variants in Chinese OI cohort.

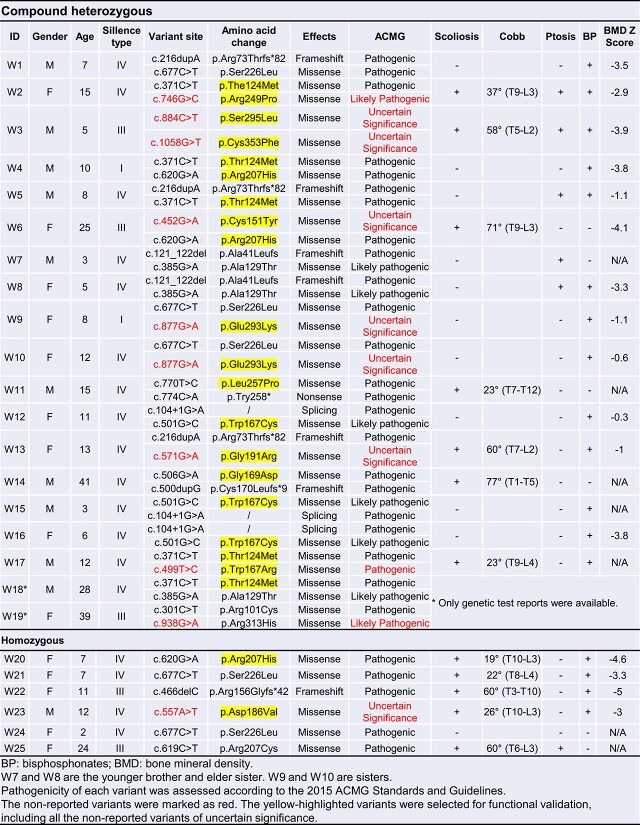

As the functional impacts of the missense or splicing variants on the WNT1 activity and patient bone phenotypes were unknown, we conducted functional assays to examine the molecular consequences of *WNT1* variants with uncertain functions. Three patients (W12, W15, and W16) were identified harboring the same splicing variant (c.104+1G>A) (Table 1). We then confirmed its impact on the splicing of *WNT1* mRNA by minigene assay (Figure 1B). The PCR product obtained from mutant group was significantly shorter than the WT band. Sanger sequencing of the PCR products revealed a 110-bp deletion at the 3′ end of exon 1 (Figure 1B). Notably, the original *WNT1* ATG (start codon) was located inside this region. The c.104+1G>A variant led to an alteration of translational starting site and disruption of WNT1 proteins.

### Compromised WNT signaling activities caused by *WNT1* variants

**Figure 2:**
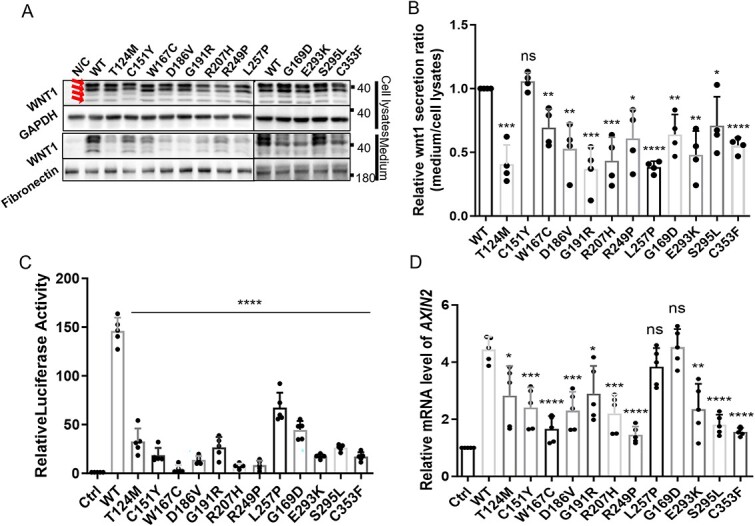
Functional analyses of WNT1 variants. (A) Western blot showing WNT1 expression and secretion in MC3T3-E1 cells. Cell lysates and culture medium were collected from MC3T3-E1 cells with WNT1 overexpression. GAPDH and fibronectin were used as endogenous controls for cell lysate and culture medium, respectively. Four intact shifting bands were indicated with red arrows on the left. (*B*) Quantification of WNT1 secretion levels among WT and mutant forms. The final WNT1 secretory capacity was represented by the ratio of secreted levels to cell lysate levels. The results were means of 4 independent experiments. The band intensity of WT was normalized to 1. (C) Dual luciferase reporter assay to assess the WNT signaling activities induced by WT and mutant WNT1 proteins using HEK293T cells. The graph showed averaged results from 5 independent experiments. (D) Quantitative PCR showed induction of canonical WNT signaling in mRNA level, by measuring transcription of downstream target gene *Axin2*. The results were averaged from 5 independent experiments. (Data were shown as means ±SD. *p*-values were calculated by comparing to WT using Student’s *t*-test, 2-tailed, unpaired. ^*^p<.05, ^**^p<.01, ^***^p<.001, ^****^p<.0001.)

We next explored the deleterious impacts of *WNT1* variants on its molecular function using the osteogenic cell line MC3T3-E1. To trigger the signaling cascade and elicit downstream effects, secreted WNT1 functions in paracrine or autocrine manners. We first analyzed the secretory capabilities of WNT1 in various mutant forms. Compared to the blank group (N/C), obvious amounts of WNT1 proteins could be detected after transfection with WT and mutant vectors (Figure 2A). Four intact shifting bands were detected on the blots, suggesting post-translational N-glycosylation partially required for WNT1 secretion.^27^ To quantify the secretion ratios of various WNT1 proteins, cytoplasmic WNT1 levels were normalized by GAPDH from cell lysate and secreted WNT1 levels were normalized by fibronectin from culture medium. Most variants significantly affected the secretion capability of WNT1 except for p.C151Y and p.S295L (Figure 2B). WNT1 functions as an important ligand to activate WNT/β-catenin signaling pathway.^28^ We applied dual-luciferase reporter assay and qRT-PCR to measure WNT signaling activities induced by different mutant forms of WNT1. To avoid the endogenous WNT signaling activity in MC3T3-E1 cells, we conducted the luciferase assay in the HEK293T cells. Compared with the WT WNT1, the transactivation function was severely compromised with different amino acid substitutions (Figure 2C). Axin2 is a critical target gene of β-catenin-dependent WNT signaling.^29^ We measured the transcription level of *Axin2* to examine the cellular impact of WNT1 variants. Consistent with the luciferase assay, variants including p.G169D and p.L257P still maintained partial transactivation activity of WNT1. Other variants significantly reduced the mRNA level of *Axin2* (Figure 2D).

### Clinical and molecular diversification in type XV OI cohort

**Figure 3:**
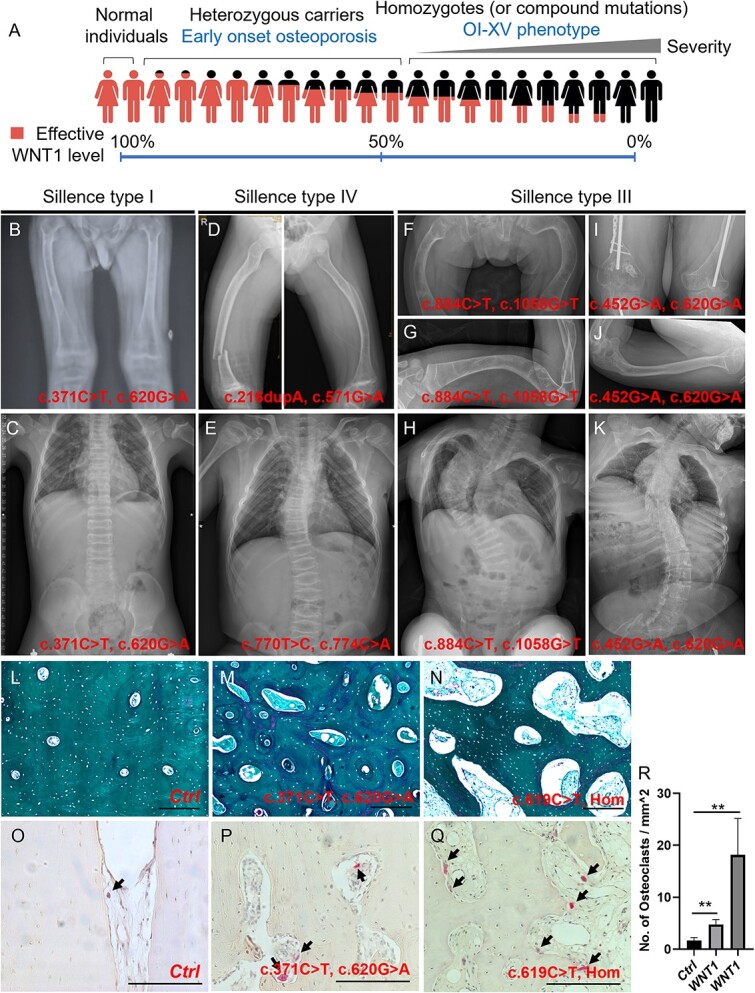
Clinical phenotypes and representative bone histology in patients with *WNT1* variants. (A) Hypothetical model showing the potential correlation of effective WNT1 level and phenotypic variations observed in normal and carriers with compound heterozygous and homozygous *WNT1* variants. (B–K) Representative cases of OI-XV. Patients were categorized into mild (B and C, Sillence type I), moderate (D and E, Sillence type IV), and severe (*F–K*, Sillence type III) types based on the bone density, deformity, frequency of fracture, Cobb angle of scoliosis, and healing performance. The variants of *WNT1* were shown respectively. (L–N) Haversian structure was shown by Goldner trichrome staining on the transaxial sections of skeletal samples from control individual (L, healthy male, 14 yr old) and patients with *WNT1* variants (M: c.371C>T, c.620G>a; N: c.619C>T, Hom). (O–Q) Representative images of tartrate resistant acid phosphatase (TRAP) staining of bone sections from the same individuals in the upper panel, respectively. Variants in *WNT1* alleles were indicated at the bottom with TRAP-positive osteoclasts arrowed. Scale bar = 100 μm. (R) Quantification of TRAP^+^ osteoclasts on the bone sections from the same individuals in *O–Q*. More than 3 nonconsecutive sections in each individual were quantified and the averaged data with standard deviations were presented in bar charts. *p*-values were calculated by Student’s *t*-test, 2-tailed, unpaired. ^*^^*^p-value <.01.

Heterozygous and homozygous variants in *WNT1* caused mild and severe skeletal syndromes, respectively,^6^ thus the secretion capacity and activity of WNT1 might be important for its downstream functions (Figure 3A). To build up more connections between genetic variants and clinical features, we summarized the clinical features and bone architecture of type XV OI patients (Table 1). Most patients showed severe low bone mass (11 patients: Z score < −2.0; 5 patients: −2.0 < Z score < 0; 9 patients without BMD records) and 19 patients reported bisphosphonate treatment histories. According to the Sillence classification,^5^ our cohort displayed predominantly moderate to severe deformities, with 2 (8%), 18 (72%), and 5 (20%) patients classified as type I (mild deformity, Figure 3B and C), IV (moderate limb deformity with Cobb angle < 45°, Figure 3D and E) and III (obvious limb and spine deformity with Cobb angle >45°, Figure 3F–K), respectively. Twelve patients (48%) developed scoliosis with Cobb angles ranging from 19° to 77°. Six patients (24%) presented ptosis (Table 1).

The mechanical strength and skeletal deformities are tightly associated with the collagen alignment and bone microstructure. To investigate the underlying molecular basis correlated with the severity of type XV OI patients, we characterized the bone geometry in the control and type XV individuals. Transaxial sections showed compact haversian structure in the control sample (healthy male, 14 yr old, fracture) (Figure 3L), whereas the size of haversian canals and resorption cavities was significantly increased in the cortical bones from type XV OI patients (Figure 3M and N), suggesting that WNT1 variants exerted deleterious effects on the bone geometry. Bone homeostasis is maintained by bone-forming osteoblasts and bone-resorbing osteoclasts. WNT signaling promotes osteogenesis but inhibits osteoclast differentiation.^30^ Thus, we examined the difference of osteoclasts between the control and mutant samples by TRAP staining. Significant more TRAP^+^ osteoclasts were detected in the bone cavities from patients with *WNT1* variants, when comparing to the normal compact bone (Figure 3O–R).

### Proteomic analysis highlighted significant downregulation of SOST in type XV OI

**Figure 4:**
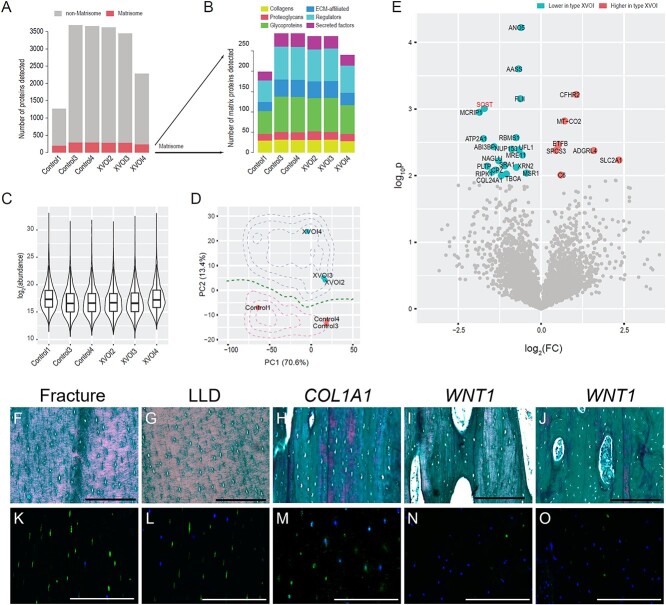
Proteomic analyses from type XV OI and control samples. (A) Bar-chart showing the numbers of proteins detected per sample. Red: matrix proteins (matrisome); gray: other proteins. Tibia/femur samples were collected from Control1: *COL1A1* c.858+1G>A, male, 14 yr old; Control3 & Control4: *COL1A1* c.4014T>A, p.Tyr1338^*^, male, 12 yr old; XVOI2: *WNT1* c. 619C>T, homozygous, female, 23 yr old; XVOI3: *WNT1* c.677C>T, c.877G>A, female, 9 yr old; XVOI4: *WNT1* c.121_122del, c. 385G>A, male, 2 yr old. (B) Bar-chart showing the numbers of each category of matrisome proteins detected. (C) Violin plot showing the protein expression levels in logarithmic scales. (D) Scatterplot showing the principal component analyses (PCA) of the 6 proteomic samples based on the proteins expressed in all samples. The contours showed the probability densities based on logistic regression results. (E) A volcano plot showing the differentially expressed proteins between the type XV OI and control samples. *p*<.01 was considered significant. (F–J) Bone histology and collagen matrix alignment were analyzed by Goldner trichrome staining on sagittal sections of skeletal samples from fracture individual (F, healthy male, 14 yr old), patient with leg-length discrepancy (G, male, 7 yr old), patient with *COL1A1* frameshift variant (H, *COL1A1* c.4014T>A, p.Tyr1338^*^, male, 12 yr old), patients with *WNT1* variants (I: *WNT1* c.619C>T, Hom, female, 23 yr old; *J*: *WNT1* c.371C>T, c.620G>A, male, 8 yr old). (K–O) Immunostaining of SOST on bone sections from the same individuals in the upper panel respectively. Scale bar = 100 μm.

Bone tissues comprised inorganic (calcium and phosphate) and organic (collagens and other extracellular matrix) materials. To understand the impact of WNT1 variants on the bone signature, we performed liquid-phase chromatography spectrometry proteomics on bone tissues isolated from controls (OI patients with splicing or frameshift variants in *COL1A1*: Control-1, 3 and 4) and type XV OI patients (XVOI-2, 3 and 4). We detected 199, 293, 293, 286, 286, and 240 matrisome (extracellular matrix) proteins in Control-1, Control-3, Control-4, XVOI-2, XVOI-3 and XVOI-4, respectively; and 1065, 3391, 3364, 3338, 3166, and 2046 non-matrisome proteins in these samples (Figure 4A). No difference was found in the numbers of matrisome (*t*-test *p* = .81) nor non-matrisome proteins (*p* = .80) between the control and case groups. We further broke down the matrisome into core (including collagens, proteoglycans, and glycoproteins) and non-core matrisome (including Extracellular matrix (ECM)-affiliated, ECM regulators, and secreted factors) (Figure 4B). We found that 28–32 collagens were consistently detected in each sample, while other core matrisomes were more variable, with 16–21 proteoglycans and 56–87 glycoproteins detected per sample. Around 23–42 ECM-affiliated proteins, 53–81 regulators, and 21–33 secreted factors were identified in each sample. None of these matrisome categories displayed higher preference in the case or control groups (Figure 4B). We confirmed that the protein abundances in logarithmic scale were comparable among all the samples (Figure 4C). Principal component analysis (PCA) using the 1154 proteins expressed in all 6 samples showed that the control samples were separable from the case group (Figure 4D), suggesting that subtle molecular differences existed between the 2 groups. To detect the underlying differences, we performed proteome-wide differentially expressed protein detection and found 20 downregulated and 6 upregulated proteins in the case group (Figure 4E). Of note, SOST (sclerostin, an osteocyte-derived WNT inhibitor) was among the most downregulated proteins. To validate, we performed immunostaining of SOST in a set of 5 independent samples. Mature osteocytes were embedded in well-organized bone matrix with a spindle-shaped canalicular network in the control bones (Figure 4F and G, patients with fracture and LLD). The densities of osteocytes were comparable in these 2 samples (Fracture: 1535 ± 266 osteocytes/mm^2^; LLD: 1499 ± 136 osteocytes/mm^2^; *p* = .41). The collagen alignment and osteocyte morphology were relatively normal in the patients with *COL1A1* frameshift variant (c.4014T>A, p.Tyr1338^*^) (Figure 4H). On the other hand, the collagen alignment and osteocyte morphology became irregular in type XV OI samples (Figure 4I and J). Immunostaining showed that SOST signal was depleted in the OI samples with *WNT1* variants, while it was uniquely expressed in mature osteocytes (Figure 4K–O). The downregulation of SOST suggested that the bone formation was compromised with aberrant osteocyte maturation in the type XV OI patients.

### Single-cell transcriptomics revealed aberrant differentiation trajectory of skeletal progenitors in type XV OI bone

To probe the cellular and molecular differences in the skeletal system, we performed single-cell transcriptomics (scRNA-seq) on the tibia specimens isolated from a type XV OI patient (male, 8 yr old) carrying compound heterozygous variants in *WNT1* (c. 371C>T, p.T124M and c. 620G>A, p.R207H) and a patient with LLD (male, 7 yr old). The patient with LLD showed normal bone shape and mineral density (Figure S1). The bone morphology and SOST expression profile were comparable with normal individuals (Figure 4F and G, K and L). Thus, we used it as a reference for the scRNA-seq analyses.

**Figure 5:**
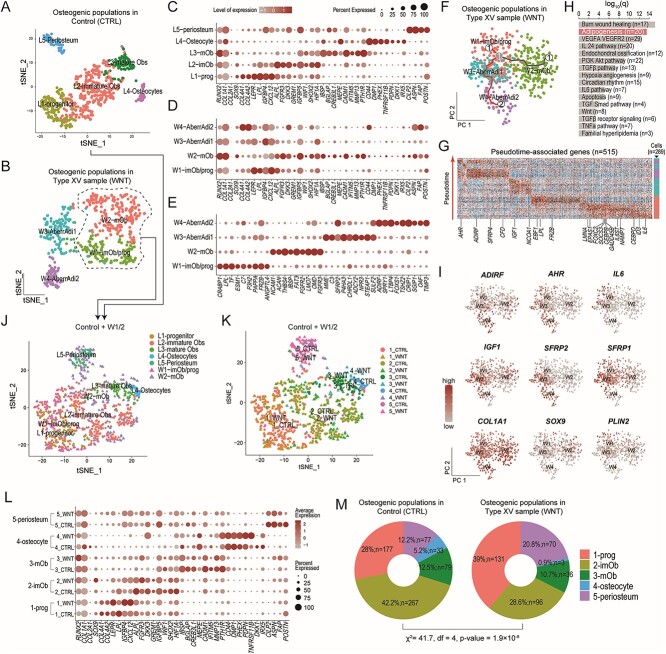
Single-cell transcriptomic analyses. (A) Scatterplot showing the dimension reduction results by tSNE on the osteogenic cells of the control sample (tibia, 7 yr old, male). (B) Scatterplot showing the dimension reduction results by tSNE on the osteogenic cells of the type XV OI sample (tibia, 8 yr old, male). (C) Dot-plot showing the expression levels and percentages of top signature genes for each subpopulation identified in the control sample. For reference, *RUNX2*, *COL1A1*, *COL2A1*, and *SOX9* were also shown. (D) Dot-plot showing the expression levels and percentages of the same set of genes in (C) for each subpopulation of the type XV OI sample. (E) Dot-plot showing the expression levels and percentages of top signature genes for each subpopulation identified in the type XV OI sample. (F) Scatterplot showing the pseudotime trajectories of the subpopulations in the type XV OI sample predicted by Monocle3. Two branches were detected starting from W1-imOB/prog, one heading toward mature osteoblasts (W2-mOb) and the other heading toward the W3 and W4 subpopulations (W3-AberrAdi1, W4-AberrAdi2). The cells were color-coded by subpopulations. (G) Heatmap showing the 515 pseudotime-associated genes for the W4 branch in (F). The 20 adipogenesis genes in (H) were highlighted. (H) Bar-chart showing the enriched canonical pathways. Adipogenesis was highlighted. (I) A panel of scatterplots showing the expression levels of key adipogenesis genes detected in (H) and (G). (J) Scatterplot showing the integration of the control sample and the W1 and W2 subpopulations in the type XV OI sample. (K) The same scatterplot in (J) but with newly identified clusters. (L) Dot-plot showing expression patterns of those signature genes (C) in the integrated and re-clustered subpopulations. (M) Donut plots showing the proportions of the 5 subpopulations in each sample.

After quality assessment using a standard protocol, 7605 and 5890 cells from the control (CTRL) and case (type XV OI) samples were collected, respectively (Figure S2A–H). After clustering and signature identification, leukocytes (*CD45^+^*), smooth muscle cells (SMC, uniquely expressing *TAGLN*, *NOTCH3*, *MYH11*, etc.), endothelial (uniquely expressing *CDH5*, *PECAM1*, *CD34*, etc.), and osteogenic (uniquely expressing *RUNX2*, *BGLAP*, *COL1A1/2*, etc.) clusters were detected in both samples (Figure S2A–H). In particular, 633 and 528 osteogenic cells were identified in the control (CTRL) and case samples (WNT), respectively (Figure 5A and B). We then isolated the osteogenic cells from both samples for further analyses. In the control sample, 5 clusters were identified, including osteoprogenitors (L1-prog, 149 cells, expressing *COL4A1/2*, *LEPR*, *IGFBP4*, *CXCL12*), immature osteoblasts (L2-imOb, 297 cells, expressing *ALPL*, *FGFR3*, *DKK3*, *GREM1*, *WIF1*), mature osteoblasts (L3-mOb, 82 cells, expressing *IBSP*, *BGLAP*, *CREB3L1*, *MEPE*, *CADM1*, *IFITM5*, *MMP13*, *PTH1R*), osteocytes (L4-Osteocyte, 38 cells, expressing *CD44*, *DMP1*, *PHEX*, *TNFRSF11B*, *PDPN*, *IRX5*), and periosteum (L5-periosteum, 67 cells, expressing *CILP2*, *ASPN*, *POSTN*) (Figure 5A; Table S1). To test whether the control sample showed normal differentiation trajectory, we compared the human subpopulations with mouse tibia scRNA-seq data at P6 stage (equivalent to human 3–10 yr old)^25^ (Table S2). The comparison confirmed that osteogenic subpopulations from the control sample largely resembled those from normal mouse tibia, and thus may serve a reasonable reference.

In a similar fashion, we performed analyses on the 528 osteogenic cells from the WNT sample. Four subpopulations were identified, including W1-imOb/prog (immature osteoblasts and osteoprogenitors, 131 cells), W2-mOb (mature osteoblasts, 205 cells), W3-aberrAdi1 (aberrant cell group 1, 130 cells), and W4-aberrAdi2 (aberrant cell group 2, 62 cells) (Figure 5B; Table S1). In the control sample, all osteogenic subpopulations expressed *RUNX2*, *COL1A1*, but barely expressed *COL2A1* nor *SOX9* (Figure 5C), confirming their osteogenic identities. In the WNT sample, W1-imOb/prog and W2-mOb molecularly behaved much the same as L1-prog/L2-imOb and L3-mOb from the control sample, by expressing similar signature markers (*LEPR*, *IGFBP4*, *CXCL12* for W1 and *ALPL*, *DKK3*, *IGFBP5* for W2; Figure 5D). W3 and W4 did not seem to resemble the typical osteogenic linages from osteoprogenitors to osteocytes (Figure 5D). Consistent with the dimension reduction diagrams, they deviated and formed disjoint clusters (Figure 5B). Further analyses on the signature genes showed that W3 uniquely expressed *MME* (*CD10*), *SFRP2*, and W4 uniquely expressed *ADIRF*, *LTBP4* (Figure 5E). Of note, *ADIRF*, the top gene in W4, encodes adipogenesis regulatory factor that is adipocyte-specific.^31^ However, the W3 and W4 populations may not function as typical adipocytes, given their mixed molecular signatures (Figure 5E) that are distinct from known adipogenic lineages.^32^

To delineate the cellular and molecular signature of these cell populations, we performed pseudotime trajectory on the osteogenic cells from the WNT sample. Two major branches were found, one heading for mature osteoblasts and the other heading for W3 and W4 (Figure 5F). We next examined the molecular events along the adipogenic branch and performed a general linear model, where successive waves of genes could be detected along the pseudo-timeline (see Methods). We detected 515 pseudotime-associated genes (Figure 5G) and found that adipogenesis, TGFβ, WNT, and other pathways were significantly enriched (Figure 5H). Among the 20 genes associated with adipogenesis, 11 were first turned on in the W1-osteoprogenitors, including *CEBPB/D* and hypoxia factors *EPAS1*. Another 4 genes (*FRZB*, *LPL*, *EBF1* and *NCOA1*) were turned on in a later pseudotime stage of W1. *IGF1* was turned on in W3, while *IL6*, *SFRP4*, *ADIRF*, *AHR* were turned on in W4 (Figure 5I). *AHR* encodes aryl hydrocarbon receptor, which regulates lipid metabolism and promotes obesity with IL-6.^33^

Next, we compared the osteogenic subpopulations W1 and W2 (*n* = 336 cells) with those in the control sample. After aggregation, 5 clusters were identified, corresponding to prog, imOb, mOb, osteocyte, and periosteum (Figure 5J; Figure S2I–K; Table S1). Interestingly, although no distinct osteocyte or periosteum subpopulations were identified in the original WNT sample (Figure 5B), perhaps due to the limited cell number, they were detected in clusters 4 and 5 after being delineated by their original sample sources (Figure 5K), respectively. These osteocytes and periosteum cells in WNT sample exhibited highly similar molecular characteristics as the control counterparts (Figure 5L). Proportion-wise, we found that there were more progenitors, but fewer mature Obs and osteocytes in the WNT sample (χ^2^  *p* = 1.9×10^-8^) (Figure 5M; Figure S2L). In particular, there were only 3 osteocytes (0.9%) in the WNT sample, as compared with 33 osteocytes (5.2%) in the control (Figure 5M). Taken together, these results showed that more immature progenitors and fewer mature osteoblasts and osteocytes were found in the type XV bone tissues and aberrant osteogenic cell populations with adipogenic characteristics were present.

### WNT1 promotes osteogenesis but inhibits adipogenesis

**Figure 6:**
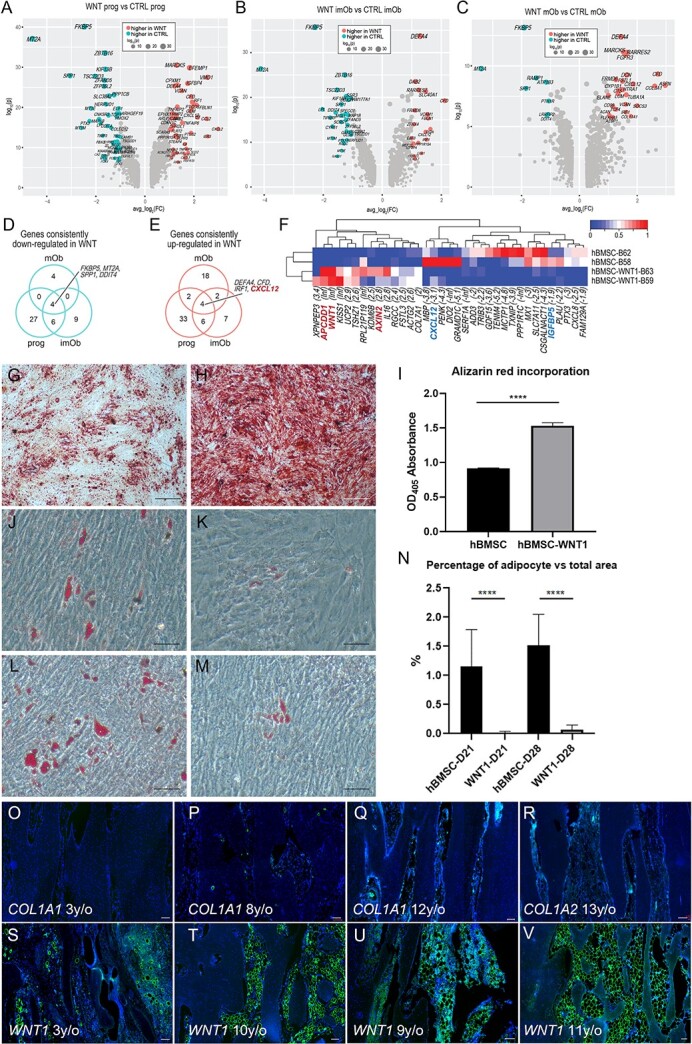
WNT1 promotes osteogenesis but inhibits adipogenesis. (A) Volcano plot showing the differentially expressed genes (DEGs) between the progenitor cells from type XV OI and control samples. (B) Volcano plot showing the DEGs between the immature osteoblasts from type XV OI and control samples. (C) Volcano plot showing the DEGs between the mature osteoblasts from type XV OI and control samples. (D and E) Venn diagrams showing the overlaps between the downregulated (D) and upregulated (E) genes in the 3 subpopulations. (F) Heatmap showing the 35 DEGs between the hBMSC and hBMSC with WNT1-overexpression. (G and H) Alizarin red staining of hBMSC (G) and hBMSC overexpressing *WNT1* (H) at D14 after induced by osteogenic medium. (I) Alizarin red staining rate quantified by OD_405_ absorbance. (J–M) Oil red staining of hBMSC (J and L) and hBMSC overexpressing *WNT1* (K and M) at D21 (J and K) and D28 (L and M) after induced by adipogenic medium. (N) Cells positive with oil red staining were quantified. Data were shown as means ±SD (*n* = 3 for each sample; asterisks denote statistical significance: ^*^^*^^*^^*^p<.0001 compared to control group). (O–V) Immunostaining of Perilipin on bone sections from patients with type I collagen variants (O: *COL1A2*, c.2943delT, p.G982Vfs^*^40; P: *COL1A1*, c. 2299G>A, p.G767S; Q: c.4014T>A, p.Tyr1338^*^ (proteomic sample); R: *COL1A2*, c. 1045G>A, p.G349S) and patients with *WNT1* variants (S: *WNT1*, c.121_122del, p.A41fs113 and c. 385G>A, p.A129T; T: *WNT1*, c. 371C>T, p.T124M and c. 620G>A, p.R207H; U: *WNT1*, c.677C>T, c.877G>A (proteomic sample); V: *WNT1*, c.466delC, p.R156Gfs^*^42). (y/o: years old). Scale bar = 100 μm.

In addition to the proportional changes in the osteogenic subpopulations, and aberrant cell populations found in the WNT sample, we further explored the underlying molecular mechanism by detecting the differentially expressed genes among each osteogenic subpopulation in the WNT and control samples, except for the osteocytes due to the limited cell number. Forty-five DEGs higher and 47 lower in the WNT osteoprogenitors were detected as compared with the control (Figure 6A). The upregulated genes included matrix genes (*VCAN*, *EFEMP1*, *FBLN1*) and reported osteoprogenitor markers *STEAP4*^25^ and *CXCL12.*^34^ The downregulated genes included osteoblast markers *SPP1* (Osteopontin), *POSTN*, and *TNC*. Similarly, we detected 19 DEGs higher and 29 lower in the immature osteoblasts (Figure 6B); and 26 DEGs higher and 8 lower in the mature osteoblasts (Figure 6C) from the WNT sample as compared with the control. Venn diagram showed that 4 genes were consistently downregulated in all comparisons, including *SPP1*, *FKBP5*, *MT2A*, and *DDIT4* (Figure 6D), and 4 genes were consistently upregulated in all WNT subpopulations, including *DEFA4*, *CFD*, *IRF1*, and *CXCL12* (Figure 6E). *CXCL12* is a typical marker of mesenchymal progenitors, and descendants of *CXCL12* are able to differentiate into osteoblasts.^35^^,^^36^ The upregulation of *CXCL12* in the osteogenic subpopulations of WNT sample suggested the differentiation of osteoprogenitors may be compromised.

To investigate the impact of WNT1 on the differentiation of mesenchymal progenitors, we overexpressed *WNT1* in human BMSC and performed bulk transcriptomics (*n* = 2 for each group). We detected 35 DEGs, including 14 higher and 21 lower in the overexpressed samples (Figure 6F). The upregulated genes included *WNT1* and *AXIN2*, a key member of canonical WNT pathway, confirming the validity of the overexpression experiment. Most interestingly, the downregulated genes included *IGFBP5* and *CXCL12*, both of which are markers of osteoprogenitors. These data suggest that WNT1 may prime the differentiation potential of mesenchymal progenitors. To further test, we compared the differentiation ability of hBMSC and hBMSC-WNT1 toward osteogenesis and adipogenesis. After 14 d of osteogenic induction, we observed more calcium deposition in hBMSC transfected with *WNT1* (Figure 6G–I), indicating higher osteogenic potential, which was consistent with the previous finding.^13^ On the other hand, WNT1 overexpression inhibited adipogenesis of hBMSC, as very little oil droplets were produced after 21 and 28 d of adipogenic induction compared to WT hBMSC (Figure 6J–N). Finally, we examined the impact of WNT1 loss-of-function on the differentiation potential of skeletal progenitors. In the cortical bones from OI patients with *COL1A1/A2* variants, only a few adipocytes were detected in the cavities (Figure 6O–R). However, adipocytes were significantly increased in the cortical bones isolated from type XV OI patients (Figure 6S–V). These results suggested that WNT1 promoted osteogenesis but inhibited adipogenesis during bone development, growth, and maintenance. WNT1 loss-of-function may result in aberrant differentiation trajectory of skeletal progenitors in type XV OI, leading to excessive *CXCL12^+^* progenitors/immature osteoblasts and abnormal cell populations with adipogenic characteristics.

## Discussion

The patients with *WNT1* variants mainly presented moderate (type IV) to severe (type III) symptoms, while those with *COL1A1* and *COL1A2* variants in our cohort were predominantly types I and IV,^12^^,^^21^ consistent with the concept that autosomal recessive OI is generally associated with more severe phenotypes.^7^ Although Wnt1 is crucial for brain development,^9^ only 6 patients in our cohort (6/25) showed ptosis that is related to neuronal defects (Table 1). In a previous Chinese cohort study involving 16 patients diagnosed with type XV OI, congenital cerebral dysgenesis was reported in one patient,^12^ while all the patients in our cohort develop with normal intelligence. The cross regulation in the nervous and skeletal systems by WNT1 remains largely unknown. Three SNPs in the *WNT1* locus were identified significantly associated with BMD and fracture in the GWAS of 426 824 participants from the UK Biobank,^37^ further confirming its essential role in bone homeostasis. Previous studies and our panel sequencing data suggested that *WNT1* represents the most frequent pathogenic gene in the recessive form of OI in Chinese population.^7^ Given that *WNT1* heterozygous variants caused dominantly inherited early onset osteoporosis,^6^ it is worthy to explore the impact of *WNT1* variants in the osteoporotic population of Chinese ethnic.

The structures of 2 WNT proteins, Xenopus XWnt8 and Drosophila WntD, have been fully elucidated, providing more in-depth insights into evolution and functions of WNT family.^38^^,^^39^ The structure of WNT1 protein predicted by AlphaFold database (Figure S3) showed highly similar structure with XWnt8,^40^ which could provide a clear concept of compositions and variant distribution on WNT1 protein. Our mutagenesis assay implied the detrimental properties of the variants on the binding affinity and triggering activities of WNT1. From the results of western blot, variants of p.T124M, p.G191R and p.L257P may impair the trafficking and secretion of WNT1, while p.C151Y possibly affects WNT1 activity through weakening its binding to Frizzled receptor, as the tyrosine substitution may disrupt the disulfide bond between Cysteine143 and Cysteine151 that is required for active WNT ligands and signal transduction.^40^

Previous studies have shown that WNT1 regulates the development of central nervous system and bone homeostasis via canonical β-catenin pathways.^12^^,^^15^^,^^41^ Recent studies suggest that noncanonical pathways like the mTOR pathway are also involved to mediate the function of WNT1.^13^ Our bulk transcriptomic data indicated that canonical WNT pathway was activated with WNT1 overexpression. The spatial expression pattern of WNT1 in the skeletal tissues remains unclear. Lineage tracing experiment showed that osteocytes were descendants of *Wnt1-Cre* expressing cells.^6^ However, we did not detect the *WNT1* expression in the osteogenic population, which may be due to the low expression of *WNT1* or the inadequate sequencing depth of 10×single-cell transcriptome.

High level of WNT signaling is required for the induction of osteogenesis via Runx2^42^ and inhibition of osteoclast differentiation by cAMP/PKA pathways.^43^ However, a previous study revealed reduced osteoclast number on the bone surfaces in type XV OI patients, though they showed enhanced bone density after treatments with bisphosphonates and denosumab, which inhibit the function of osteoclasts.^12^ Our data from human samples confirmed that reduced osteogenesis but enhanced osteoclast activities were observed in the type XV OI patients. It is still unknown whether the bisphosphonates have any side effects on the skeletal lineage. The bisphosphonate treatments were discontinued at least 3 mo before and after the osteotomies to minimize its effects on the osteogenic cells. The single-cell transcriptome and proteomic data also indicated that the maturation of osteocytes was impaired with WNT1 loss-of-function. Treatment with anti-SOST antibody is one of the options to improve bone mass in OI patients. However, the expression level of SOST was significantly downregulated in type XV OI patients, which may explain the compromised effect of Sclerostin antibody in treating *Wnt1* swaying mice.^13^

Canonical WNT signaling inhibits adipogenesis in white adipose tissue. Removal of β-catenin in pre-osteoblasts shifts its cell fate from osteoblasts to adipocytes.^44^  *In vitro* differentiation assays confirmed the anabolic effect of WNT1 in osteogenesis and inhibitory function in adipogenesis. What’s more, in the single-cell RNA-seq analyses, abnormal cell populations identified in the WNT sample showed characteristics of adipocytes. Among the 4 genes upregulated in all WNT subpopulations (*DEFA4*, *CFD*, *IRF1*, and *CXCL12*), both *CFD*^45^ and *IRF1*^46^ were known to be regulating bone homeostasis, while *DEFA4*^47^ and *CXCL12* were markers of the stromal cells. Descendants of *CXCL12* were known to differentiate into osteoblasts.^35^ Interestingly, *CXCL12* is negatively regulated by the WNT pathway in the BMSC.^48^  *CXCL12* deletion increased bone formation.^49^ It suggests that *CXCL12* functions as an osteoprogenitor marker and its excessive expression may compromise bone homeostasis. On the other hand, the top gene upregulated in the aberrant cell populations, *ADIRF*, is a master factor of adipogenesis acting upstream of PPARγ and C/EBPα,^31^ with its overexpression inducing proliferation and inhibiting apoptosis in preadipocytes.^50^

### Limitations of the study

In this study, although we summarized the clinical manifestations and genetic variants of 25 patients with type XV OI, the patient number was limited due to the scarcity of OI. We also revealed the impact of *WNT1* variants on its functional activity. However, *in vivo* testing using knock-in animal model is lacking. Although abnormal lineage trajectory was observed from the single-cell RNA-seq analyses, the control sample from a patient with LLD was diagnosed with Proteus syndrome with a mosaic variant in the *AKT1* gene (c.49G>A, p.E17K, sample frequency 6.3%–13.3%). Most of the type XV patients also received regular bisphosphonate treatments, the effects of which on the osteogenic lineage remain unclear. The underlying mechanisms how WNT1 modulates osteocyte maturation need to be consolidated by increasing the sample sizes of single cell transcriptomic (*n* = 1 for each group) and proteomic (*n* = 2 for control group and *n* = 3 for type XV OI) data.

In summary, our cohort study showed that the proportion of type XV OI patients was relatively high (~10%) in the Chinese population. Functional analyses indicated that variants on the *WNT1* locus significantly impaired its secretion and effective activity, resulting in moderate to severe bone deformities. Analyses of proteomic and single-cell transcriptomic data from human skeletal samples revealed significant reduction of SOST, impaired maturation of osteocytes, excessive *CXCL12^+^* progenitors, and abnormal cell populations with adipogenic characteristics. The integration of multi-omics data delineates how WNT1 regulates the differentiation of skeletal progenitors, giving deeper insights into the pathogenesis of type XV OI for novel therapeutic strategies. New treatments should be developed to promote osteocyte maturation and inhibit the adipogenesis for the type XV OI patients.

## Supplementary Material

WNT1_Supplementary_Figures_JBMR_20240701_zjae123

Supplemental_Table_1_scRNA-seq_population_markers_zjae123

Supplementary_Table_2_Comparison_of_human_and_mouse_osteogenic_lineage_zjae123
