# Supplementary material for: Multi-omics analyses reveal aberrant differentiation trajectory with WNT1 loss-of-function in type XV osteogenesis imperfecta
Source: J Bone Miner Res. 2024 Aug 10;39(9):1253–67. doi: 10.1093/jbmr/zjae123 (PMC11371906; doi:10.1093/jbmr/zjae123)
Supplement: WNT1_Supplementary_Figures_JBMR_20240701_zjae123 [file wnt1_supplementary_figures_jbmr_20240701_zjae123.pdf]

Fig. S1

A

B

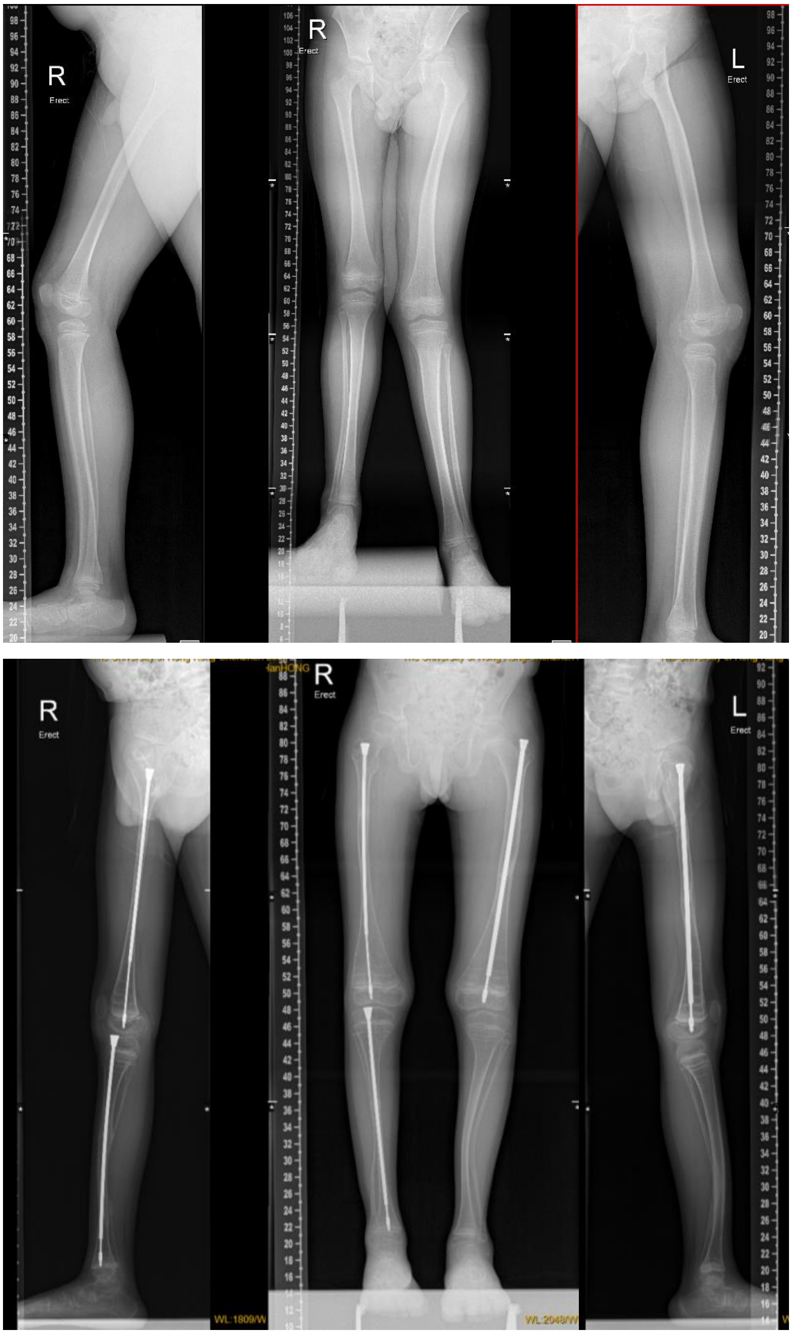

Figure S1. Clinical features of patients from which bone tissues were isolated for single-cell analyses.

(*A*) X-ray images from the patient with leg-length-discrepancy (male, 7 years old).

(*B*) X-ray images from the patient (male, 8 years old) carrying compound heterozygous variants on *WNT1* (c.C371T, p.T124M and c.G620A, p.R207H).

Fig. S2

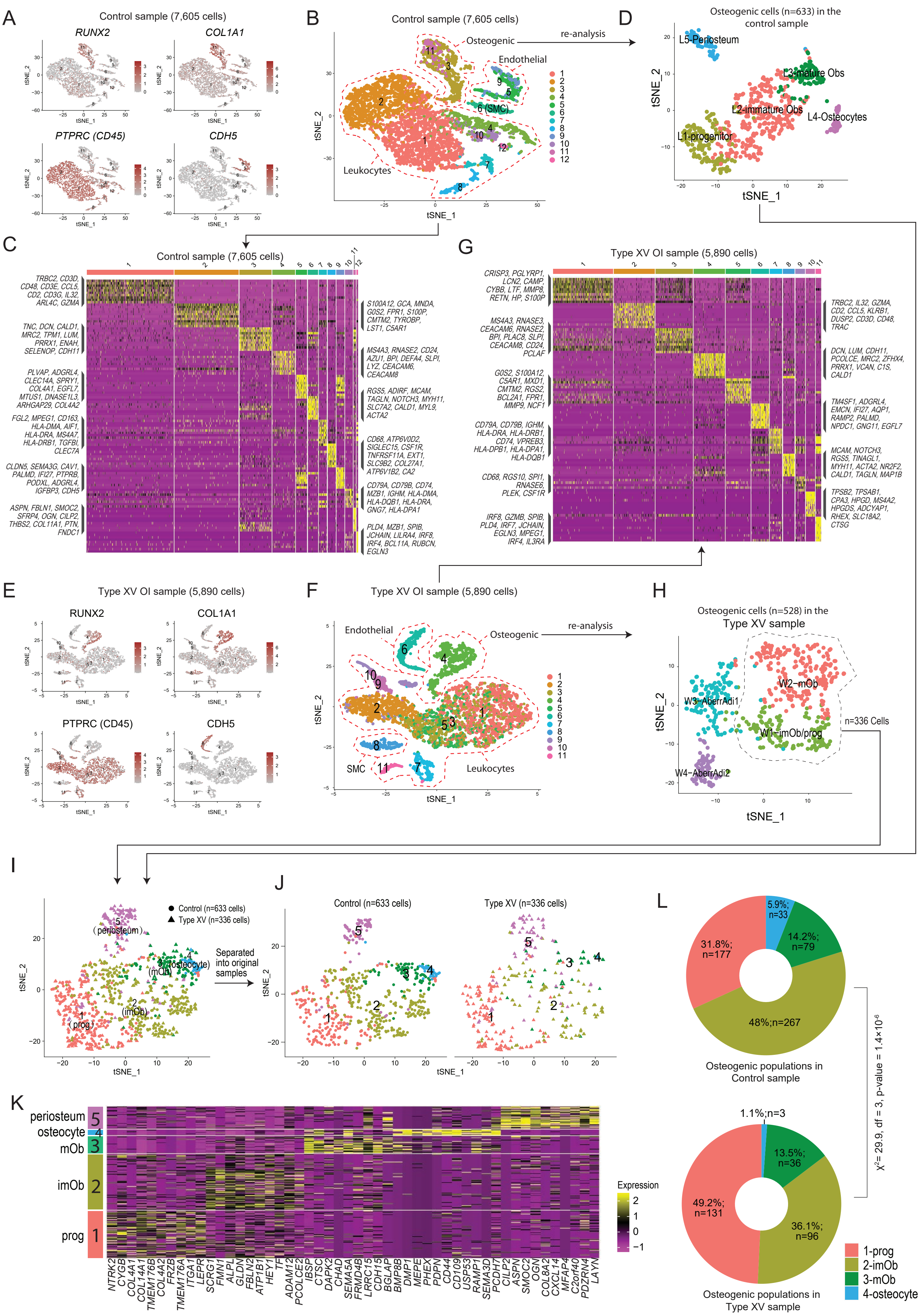

A
Control sample (7,605 cells)
RUNX2
COL1A1
PTPRC (CD45)
CDH5
B
Control sample (7,605 cells)
Osteogenic
Endothelial
6 (SMC)
Leukocytes
re-analysis
tSNE_1
tSNE_2
D
Osteogenic cells (n=633) in the control sample
L5-Periosteum
L3-mature Obs
L2-immature Obs
L4-Osteocytes
L1-progenitor
C
Control sample (7,605 cells)
TRBC2, CD3D, CD48, CD3E, CCL5, CD2, CD3G, IL32, ARL4C, GZMA
TNC, DCN, CALD1, MRC2, TPM1, LUM, PRRX1, ENAH, SELENOP, CDH11
PLVAP, ADGRL4, CLEC14A, SPRY1, COL4A1, EGFL7, MTUS1, DNASE1L3, ARHGAP29, COL4A2
FGL2, MPEG1, CD163, HLA-DMA, AIF1, HLA-DRA, MS4A7, HLA-DRB1, TGFBI, CLEC7A
CLDN5, SEMA3G, CAV1, PALMD, IFI27, PTPRB, PODXL, ADGRL4, IGFBP3, CDH5
ASPN, FBLN1, SMOC2, SFRP4, OGN, CILP2, THBS2, COL11A1, PTN, FNDC1
S100A12, GCA, MNDA, G0S2, FPR1, S100P, CMTM2, TYROBP, LST1, C5AR1
MS4A3, RNASE2, CD24, AZU1, BPI, DEFA4, SLPI, LYZ, CEACAM6, CEACAM8
RGS5, ADIRF, MCAM, TAGLN, NOTCH3, MYH11, SLC7A2, CALD1, MYL9, ACTA2
CD68, ATP6V0D2, SIGLEC15, CSF1R, TNFRSF11A, EXT1, SLC9B2, COL27A1, ATP6V1B2, CA2
CD79A, CD79B, CD74, MZB1, IGHM, HLA-DMA, HLA-DQB1, HLA-DRA, GNG7, HLA-DPA1
PLD4, MZB1, SPIB, JCHAIN, LILRA4, IRF8, IRF4, BCL11A, RUBCN, EGLN3
G
Type XV OI sample (5,890 cells)
CRISP3, PGLYRP1, LCN2, CAMP, CYBB, LTF, MMP8, RETN, HP, S100P
MS4A3, RNASE3, CEACAM6, RNASE2, BPI, PLAC8, SLPI, CEACAM8, CD24, PCLAF
G0S2, S100A12, C5AR1, MXD1, CMTM2, RGS2, BCL2A1, FPR1, MMP9, NCF1
CD79A, CD79B, IGHM, HLA-DRA, HLA-DRB1, CD74, VPREB3, HLA-DPB1, HLA-DPA1, HLA-DQB1
CD68, RGS10, SPI1, RNASE6, PLEK, CSF1R
IRF8, GZMB, SPIB, PLD4, IRF7, JCHAIN, EGLN3, MPEG1, IRF4, IL3RA
TRBC2, IL32, GZMA, CD2, CCL5, KLRB1, DUSP2, CD3D, CD48, TRAC
DCN, LUM, CDH11, PCOLCE, MRC2, ZFHX4, PRRX1, VCAN, C1S, CALD1
TM4SF1, ADGRL4, EMCN, IFI27, AQP1, RAMP2, PALMD, NPDC1, GNG11, EGFL7
MCAM, NOTCH3, RGS5, TINAGL1, MYH11, ACTA2, NR2F2, CALD1, TAGLN, MAP1B
TPSB2, TPSAB1, CPA3, HPGD, MS4A2, HPGDS, ADCYAP1, RHEX, SLC18A2, CTSG
E
Type XV OI sample (5,890 cells)
RUNX2
COL1A1
PTPRC (CD45)
CDH5
F
Type XV OI sample (5,890 cells)
Endothelial
Osteogenic
re-analysis
SMC
Leukocytes
H
Osteogenic cells (n=528) in the Type XV sample
W2-mOb
W3-AberrAdi1
W1-imOb/prog
W4-AberrAdi2
n=336 Cells
I
Control (n=633 cells)
Type XV (n=336 cells)
5 (periosteum)
4 (osteocyte)
3 (mOb)
2 (imOb)
1 (prog)
Separated into original samples
J
Control (n=633 cells)
Type XV (n=336 cells)
K
periosteum
osteocyte
mOb
imOb
prog
Expression
NTRK2 CYGB COL4A1 COL14A1 TMEM176B COL4A2 FRZB TMEM176A ITGA1 LEPR SCRG1 FMN1 ALPL GLDN FBLN2 ATP1B1 HEY1 TF ADAM12 PCOLCE2 IBSP CTSC DAPK2 CHAD SEMA5A FRMD4B LRRC15 CDH15 BGLAP BMP8B DMP1 MEPE PHEX PDPN CD44 CD109 USP53 RAMP1 SEMA3D PCDH7 CILP2 ASPN SMOC2 OGN COL8A2 CXCL14 MFAP4 C2orf40 PDZRN4 LAYN
L
5.9%; n=33
14.2%; n=79
31.8%; n=177
48%;n=267
Osteogenic populations in Control sample
1.1%;n=3
13.5%; n=36
49.2%; n=131
36.1%; n=96
Osteogenic populations in Type XV sample
χ² = 29.9, df = 3, p-value = 1.4×10⁻⁶
1-prog
2-imOb
3-mOb
4-osteocyte

Figure S2. Additional scRNA-seq analyses.
(A) tSNE plots showing the overall picture of the control sample (7,605 cells), where *RUNX2* and *COL1A1* marking the osteogenic cluster, *PTPRC* (*CD45*) marking the leukocytes, and *CDH5* marking the endothelial cells.
(B) tSNE plot showing the control sample with cluster labels.
(C) Heatmap showing the top 10 signature genes for each cluster in the control sample.
(D) Clusters of the osteogenic cells in the control sample.
(E) tSNE plots showing the overall picture of the WNT sample (5,890 cells), where *RUNX2* and *COL1A1* marking the osteogenic cluster, *PTPRC* (*CD45*) marking the leukocytes, and *CDH5* marking the endothelial cells.
(F) tSNE plot showing the WNT sample with cluster labels.
(G) Heatmap showing the top 10 signature genes for each cluster in the WNT sample.
(H) Clusters of the osteogenic cells in the WNT sample.
(I) tSNE plot showing the integrated analyses of osteogenic subpopulations from (D) and W1, W2 from (H).
(J) Two tSNE plots showing the split of the plot in (I) according to the respective sample origins.
(K) Heatmap showing the expression levels of the top 10 signature genes identified in the five clusters identified in the integrated sample in (I).
(L) Donut plots showing the proportions of individual sub-populations in the two samples excluding periosteum.

Fig. S3

A

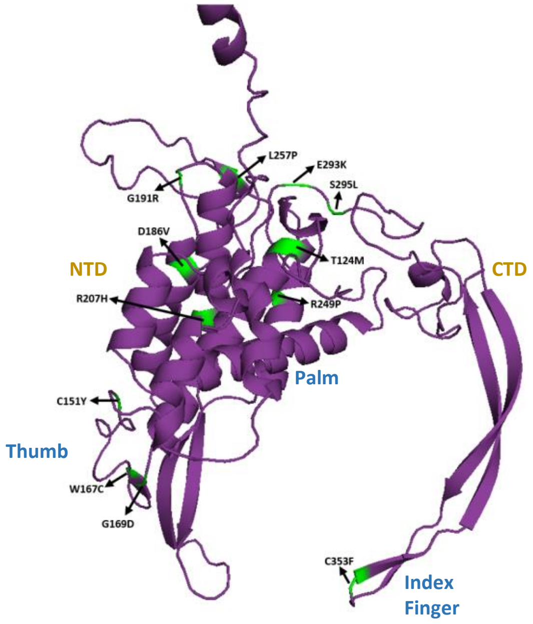

Figure S3. **WNT1 molecular models showing locations of altered amino acids.**
(*A*) Predicted ribbon model of human WNT1 protein using Alphafold Structure Database. Wnt1 molecule resembles a hand with a "palm" on the upper part, extending a "thumb" and an "index finger" toward two opposite sides for Frizzled binding. Positions of the twelve variants are highlighted in green with amino acids labeled aside. NTD, N-terminal α-helical domain. CTD, C-terminal cysteine-rich domain.
