# Supplementary material for: Multi-omics analyses reveal aberrant differentiation trajectory with WNT1 loss-of-function in type XV osteogenesis imperfecta
Source: J Bone Miner Res. 2024 Aug 10;39(9):1253–67. doi: 10.1093/jbmr/zjae123 (PMC11371906; doi:10.1093/jbmr/zjae123)
Supplement: Supplementary_Table_2_Comparison_of_human_and_mouse_osteogenic_lineage_zjae123 [file supplementary_table_2_comparison_of_human_and_mouse_osteogenic_lineage_zjae123.docx]

| **Supplementary Table 2. Comparison of single cell transcriptomic data from different clusters of human and mouse osteogenic lineages** | | | | |
| --- | --- | --- | --- | --- |
| Human osteogenic clusters | Mouse osteogenic clusters | No. of overlapping genes | Odd ratio | p-Value |
| L1-prog | Immature Obs | 87 | 11.9 | 2.5*10^-121^ |
| L2-imObs | Immature Obs | 48 | 13.6 | 3.2*10^-81^ |
| L3-mObs | Mature Obs | 54 | 11.6 | 6.8*10^-82^ |
| L4-Osteocytes | Osteocytes | 33 | 23.0 | 1.6*10^-100^ |
| Prog: progenitor; Obs: osteoblasts; imObs: immature Obs; mObs: mature Obs. | | | | |
